# Lateral Extra-articular Procedures Reduce the Risk of Revision of Anterior Cruciate Ligament Reconstruction in Elite Athletes: A Systematic Review and Meta-analysis of Comparative Studies

**DOI:** 10.1177/03635465251376655

**Published:** 2026-01-08

**Authors:** Riccardo D’Ambrosi, Alessandro Carrozzo, Edoardo Monaco, Luca Maria Sconfienza, Elmar Herbst, Mirco Herbort, Elisabeth Abermann, Christian Fink

**Affiliations:** † IRCCS Ospedale Galeazzi–Sant’Ambrogio, Milan, Italy; ‡ University of Milan, Department of Biomedical Sciences for Health, Milan, Italy; § La Sapienza University, Dipartimento di Sanità Pubblica e Malattie Infettive, Rome, Italy; ‖ Sant’Andrea University Hospital, La Sapienza University of Rome, Rome, Italy; ¶ Department of Trauma, Hand and Reconstructive Surgery, University of Muenster, Muenster, Germany; # OCM Clinic Munich, Steinerstraße, Germany; ** Gelenkpunkt-Sports and Joint Surgery, FIFA Medical Centre of Excellence, Innsbruck, Austria; †† Research Unit for Orthopaedic Sports Medicine and Injury Prevention (OSMI), Private University for Health Sciences Medical Informatics and Technology, Innsbruck, Austria; Investigation performed at IRCCS Ospedale Galeazzi–Sant’Ambrogio, Milan, Italy

**Keywords:** elite athletes, ACL, anterolateral procedures, rerupture, odds ratio

## Abstract

**Background:**

Lateral extra-articular procedures (LEAPs) have gained increasing attention as an adjunct to anterior cruciate ligament reconstruction (ACLR), particularly in individuals at high risk for reinjury. When combined with ACLR, LEAPs contribute to the restoration of normal knee kinematics and provide a significant reduction in residual anterior laxity compared with isolated ACLR. This added stability provides a protective effect on the intra-articular graft, promoting improved healing and integration while reducing mechanical stress on the reconstructed anterior cruciate ligament (ACL). As a result, these techniques have been demonstrated to result in improved performance after ACLR, higher graft survival, and lower revision rates, even in elite athletes who are at significant risk for reinjury.

**Purpose/Hypothesis:**

The aim of this study was to systematically compare the existing evidence on ACL rerupture rates by performing a meta-analysis comparing combined ACLR and LEAP versus isolated ACLR in elite athletes. The primary hypothesis of this systematic review and meta-analysis was that the addition of LEAP would reduce the rate of revision ACLR in elite athletes.

**Study Design:**

Systematic review and meta-analysis; Level of evidence, 3.

**Methods:**

The method followed the PRISMA (Preferred Reporting Items for Systematic Reviews and Meta-Analyses) guidelines. The PubMed, Embase, and Cochrane Library databases were searched to identify potentially relevant comparative studies that analyzed rerupture rate in elite athletes after isolated ACLR versus ACLR plus LEAP. The MINORS (Methodological Index for Non-Randomized Studies) score was used for quality assessment. The main outcome measure was ipsilateral ACL rerupture.

**Results:**

A total of 586 elite athletes received an isolated ACLR, whereas 417 athletes received combined ACLR plus LEAP. Rerupture was reported by 9.3% (95% CI, 5.5%-14.0%) of athletes. In the ACLR group, 14.0% (95% CI, 7.9%-21.5%) reported a rerupture, whereas in the ACLR plus LEAP group, the reinjury rate was 5.0% (95% CI, 1.2%-10.8%), with a statistically significant difference between the 2 groups (*P* = .042). Pooled odds ratio (OR) showed a 65% reduced risk of a new rupture episode in the ACLR plus LEAP group compared with the ACLR group, with an OR of 0.35 (95% CI, 0.20-0.59; *P* < .001).

**Conclusion:**

In elite athletes, adding an anterolateral procedure during ACLR significantly reduced the rerupture rate and reduced the risk of rerupture by >60%. Despite the few studies considered, our study seems to indicate that surgeons should carefully consider LEAP when treating an elite athlete in order to significantly reduce the risk of rerupture.

**Registration:**

PROSPERO: CRD42025637843.

Anterior cruciate ligament (ACL) injuries can have a significant effect on an athlete’s career, potentially shortening it and affecting individual and team performance as well as financial aspects. Despite improvements in ACL reconstruction (ACLR) techniques, rehabilitation protocols, and return-to-play criteria, graft rupture remains a critical problem in elite athletes competing in level 1 sports—high-impact activities characterized by frequent pivoting, sudden cutting, and jumping. In this population, failure rates >30% have been reported, often resulting in prolonged or incomplete return to sport.^6,32,41^

Lateral extra-articular procedures (LEAPs) have gained increasing attention as adjuncts to ACLR, particularly in individuals at high risk for reinjury.^8,15,37,38^

When combined with ACLR, LEAPs contribute to the restoration of normal knee kinematics and provide a significant reduction in residual anterior laxity compared with isolated ACLR.^12,35^ This added stability provides a protective effect on the intra-articular graft, promoting improved healing and integration while reducing mechanical stress on the reconstructed ACL.^7,22,42^

As a result, these techniques have demonstrated to result in improved performance after ACLR, higher graft survival, and lower revision rates, even in elite athletes who are at significant risk for reinjury.^13,29,33,34,37^

However, clinical evidence regarding the outcomes of elite athletes undergoing ACLR with LEAP remains limited. Therefore, a systematic review and meta-analysis is warranted to consolidate and critically evaluate the growing body of research on the efficacy of LEAPs in reducing ACL graft failure in elite athletes.^3,4,16,18^

The aim of this study was to perform a systematic review and meta-analysis of the existing evidence on ACL rerupture rates comparing combined ACLR and LEAP versus isolated ACLR in elite athletes. The primary hypothesis of this systematic review and meta-analysis was that the addition of a LEAP would reduce the rate of revision ACLR in elite athletes.

## Methods

A systematic search strategy was developed according to the PRISMA (Preferred Reporting Items for Systematic Reviews and Meta-Analyses) guidelines and is registered in the PROSPERO Registry (CRD42025637843).^26,30^

An electronic database search was performed to identify potentially relevant research articles that analyzed rerupture in elite athletes after ACLR. The MEDLINE (PubMed), Embase (Elsevier), and Cochrane Library databases were searched on February 10, 2025, and the search was repeated 2 weeks later, using the following Boolean search terms: “ACL reconstruction” OR “anterior cruciate ligament reconstruction” OR “ACL” AND “professional” OR “elite” OR “competitive” AND “anterolateral” or “extra-articular” or “ALL” or “LEAP” or “ALLR” or “Lemaire” or “Ellison” or “Cocker-Arnold.”

### Eligibility Criteria

The literature selected for this study was based on the following criteria:

#### Study Design

Only comparative studies were included in the systematic review and meta-analysis.

#### Participants and Interventions

We included comparative studies conducted in skeletally mature elite athletes who underwent isolated ACLR or ACLR plus LEAP and were evaluated for rate of rerupture. Patients undergoing concomitant procedures were not excluded if the main surgery was ACLR. An elite athlete was defined as one who is paid to perform one’s sport or one who participates in national- or international-level competitions in amateur sports, including academy players aged ≥15 years.^4^

### Type of Outcome Measures

The main extracted and recorded outcome measure was ACL rerupture, defined as a rerupture of the ipsilateral ACL.

### Data Collection and Analysis

#### Study Selection

The retrieved articles were first screened by title and, if found relevant, were further screened by reading the abstract. After excluding studies that did not meet the eligibility criteria, we assessed the entire content of the remaining articles for eligibility. To minimize the risk of bias, we reviewed and discussed all the selected articles and references as well as the articles excluded from the study. In case of disagreement among the reviewers, the senior investigator (C.F.) made the final decision. At the end of the process, additional studies that might have been missed were searched for manually by going through the reference lists of the included studies and relevant systematic reviews.

#### Data Collection Process

Data were extracted from the selected articles by the first 2 authors (R.D. and A.C.) using a computerized tool created with Microsoft Access (Version 2010). Each article was validated again by the first author (R.D.) before analysis. For each study, data on patient return to sport and rerupture rate were extracted.

#### Level of Evidence

The Oxford Levels of Evidence set by the Oxford Centre for Evidence-Based Medicine were used to categorize the level of evidence.^23^

#### Evaluation of the Quality of Studies

The quality of the selected studies was evaluated using the MINORS (Methodological Index for Non-Randomized Studies) score. The checklist includes 12 items, of which the last 4 items are specific to comparative studies. Each item was given a score of 0 to 2 points. The ideal score was set at 16 points for noncomparative studies and 24 for comparative studies.^36^

### Statistical Analysis

We performed a meta-analysis on knee rerupture events comparing isolated ACLR versus ACLR plus LEAP. We estimated the overall rerupture rate using a random-effects model. Between-study variations were assessed with the Cochran *Q* χ^2^ test for heterogeneity and the *I*^2^ statistic. Statistical heterogeneity was considered substantial if *I*^2^ > 50%.^16^ Next, we performed a subgroup analysis using a mixed-effects metaregression model with common between-study variance component across subgroups. We tested heterogeneity across groups with a Cochran *Q* χ^2^ test. Isolated ACLR was selected as reference category due to the highest number of knees within this group. The Der Simonian-Laird variance estimator for the variance and the Freeman-Tukey double arcsine outcome transformation for proportions were used in both models. For each study we calculated odds ratios (OR) and 95% CIs with isolated ACLR as reference category. A random-effects model was performed to estimate the pooled results.

## Results

**Figure 1.**
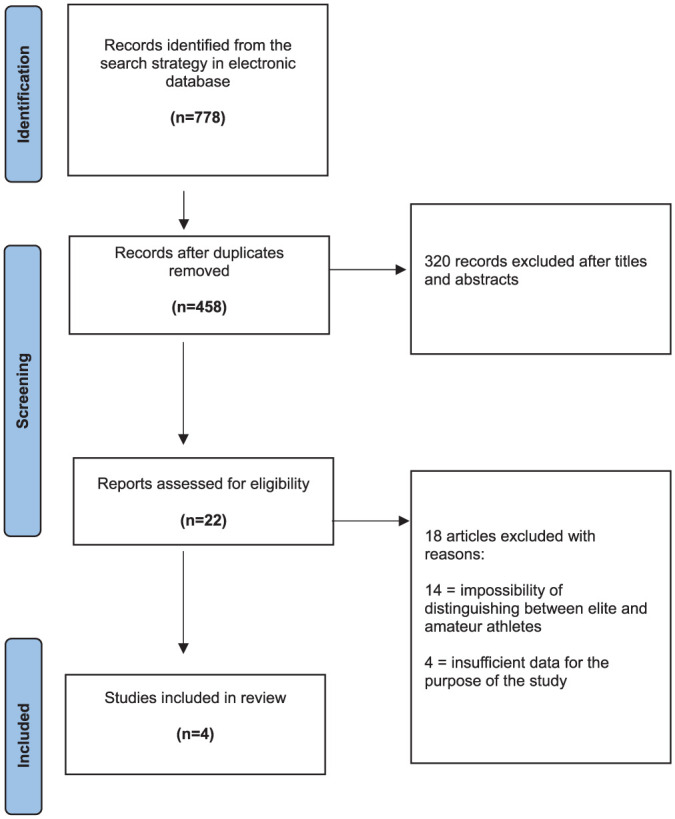
PRISMA (Preferred Reporting Items for Systematic Reviews and Meta-Analyses) flow chart indicating inclusion of research articles for final analysis.

The initial search of the 3 electronic databases yielded 778 records. The titles and abstracts of 342 studies were reviewed after elimination of 436 duplicates. After title and abstract review, 320 studies were removed, resulting in 22 full-text articles that were assessed for eligibility. Finally, the reviewers excluded 18 records after evaluating the full texts, and 4 articles were included in the final analysis of this review.^3,4,16,18^ The PRISMA diagram is shown in Figure 1.^1^

### Study Characteristics

A total of 4 comparative studies met the inclusion criteria and were included in the final analysis. All studies had a level of evidence of 3. Mean MINORS score was considered good (15 ± 0.8 points).

A total of 586 elite athletes received an isolated ACLR, whereas 417 athletes received combined ACLR plus LEAP. Details of the included studies are reported in Table 1.

**Table 1:** Patient and Study Characteristics*^a^*

					Patients, n		Sex, Male:Female, n		Age, y		
Lead Author	Year	LOE	MINORS Score	Sport, n	ACLR	ACLR+ LEAP	ACLR	ACLR+ LEAP	ACLR	ACLR+ LEAP	Follow-up
Guy^15^	2022	3	15	Alpine skiing	50	31	25:25	20:11	21.0 ± 3.9 (13-32)	24.9 ± 4.5 (19-36)	3.4 ± 3.1 y (0-13 y)
Borque^3^	2024	3	14	Rugby	88	37	114:11		23.4 ± 4.0		3.7 y
Hopper^17^	2022	3	16	Soccer, 120 Rugby, 98 Basketball, 41 Skiing, 36 Handball, 16 Motocross, 14 Other, 17	110	232	274:68		23.9 ± 5.2		100.2 ± 51.9 mo (24-215 mo)
Borque^4^	2022	3	15	Soccer, 254 Rugby, 136 Other, 65	338	117	274:64	102:15	22.9 ± 4.9	21.5 ± 4.1	≥2 y

a Age and follow-up are expressed as mean ± SD (range). ACLR, anterior cruciate ligament reconstruction; LEAP, lateral extra-articular procedure; LOE, level of evidence; MINORS, Methodological Index for Non-Randomized Studies.

#### Surgical Technique and Rehabilitation Protocol

Three studies used an anatomic procedure for ACLR, whereas only 1 study used an outside-in technique. All the authors used the modified Lemaire procedure for LEAP, whereas the authors of 2 studies performed an anterolateral ligament reconstruction (ALLR) using the hamstrings. Three studies revealed no difference in the rate of subsequent surgeries between ACLR or ACLR plus LEAP groups (*P* > .05). The rehabilitation protocol did not differ between ACLR and ACLR plus LEAP groups. Surgical and rehabilitation details are reported in Tables 2 and 3.

**Table 2:** Surgical Details of the Included Studies*^a^*

					Subsequent Surgery. n	
Lead Author	Surgical Technique	Graft	LEAP Procedure	Associated Lesions, n	Isolated ACLR	ACLR+LEAP
Guy^15^	Anatomic	BPTB Hamstring Quadriceps	Modified Lemaire with BPTB ALLR with hamstring	Medial meniscus, 24 Lateral meniscus, 35 Cartilage, 17	14*^b^*	5^b^
Borque^3^	Anatomic	BPTB Hamstring	Modified Lemaire	Medial meniscus, 21 Lateral meniscus, 42 Medial and lateral menisci, 25 ≥3 Chondral lesions, 26	Not applicable	
Hopper^17^	Outside-in	BPTB Quadriceps Hamstring	ALLR with hamstring Modified Lemaire with quadriceps or BPTB	Medial meniscus, 47 Lateral meniscus, 80 Medial and lateral menisci, 45	Medial meniscectomy, 21 Lateral meniscectomy, 7 Operation for cyclops syndrome, 13 Arthrolysis, 10 Washout for septic arthritis, 11 *^b^*	
Borque^4^	Anatomic	BPTB Hamstring	Modified Lemaire	Meniscus, 298	Subsequent surgery, 91 ^b^	

a ACLR, anterior cruciate ligament reconstruction; ALLR, anterolateral ligament reconstruction; BPTB, bone–patellar tendon–bone; LEAP, lateral extra-articular procedure.

b No difference in rate of subsequent surgery between isolated ACLR vs ACLR+LEAP.

**Table 3:** Rehabilitation Protocol of the Included Studies*^a^*

	Rehabilitation Protocol
Lead Author	Isolated ACLR and ACLR Plus LEAP
Guy^15^	Immediate weightbearing with crutches for 4 wk
	Free ROM with no brace (except in case of meniscal repair)
Borque^3^	Immediate weightbearing (for isolated ACLR)
	ROM 0°-90° for 4 wk
Hopper^17^	Brace-free, immediate full weightbearing (except in case of meniscal repairs)
Borque^4^	NA

a ACLR, anterior cruciate ligament reconstruction; LEAP, lateral extra-articular procedure; NA, not applicable; ROM, range of motion.

#### Rerupture

In total, 9.3% (95% CI, 5.5%-14.0%) of athletes reported a rerupture. In the ACLR group, 14.0% (95% CI, 7.9%-21.5%) reported a rerupture, whereas in the ACLR plus LEAP group, 5.0% (95% CI, 1.2%-10.8%) reported a rerupture, with a statistically significant difference between the 2 groups (*P* = .042).

**Figure 2.**
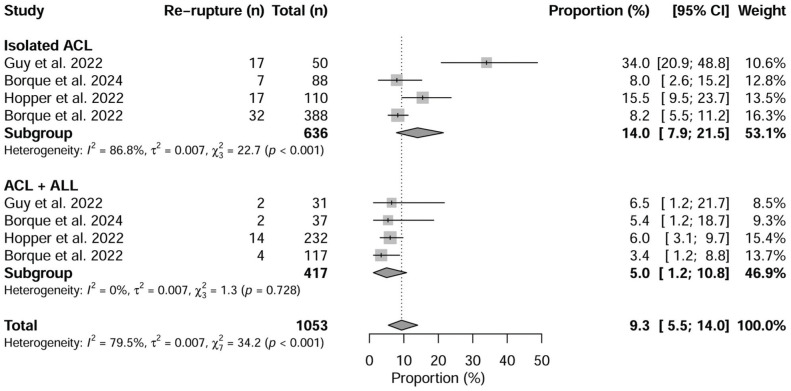
Forest plot of rerupture rate between isolated anterior cruciate ligament reconstruction (ACL) versus ACL plus lateral extra-articular procedure (LEAP).

The forest plot in Figure 2 shows the rerupture rates of the 2 groups.

**Figure 3.**
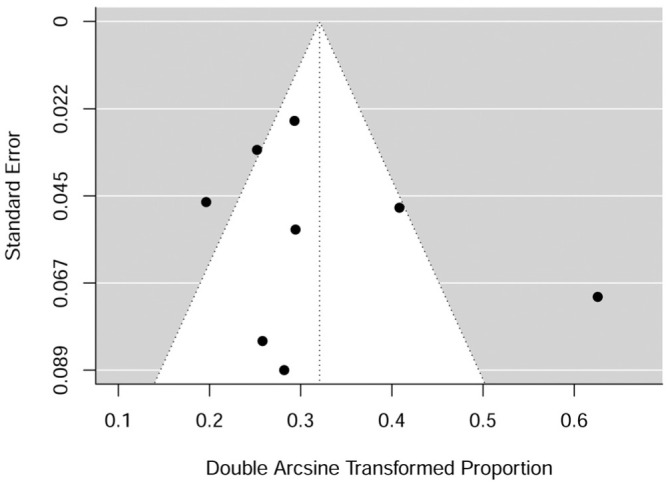
Funnel plot showing the symmetry of the included studies regarding rank correlation and linear regression on rerupture rate.

Neither the rank correlation test nor the linear regression test showed any funnel plot asymmetry (*P* = .548 and .410, respectively). A funnel plot of the estimates is shown in Figure 3.

#### Odds Ratio

**Figure 4.**
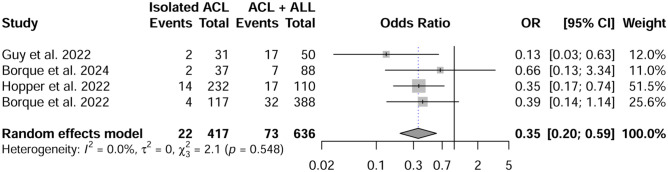
Forest plot for odds ratio for a new episode of anterior cruciate ligament reconstruction (ACL) for the 2 groups. LEAP, lateral extra-articular procedure; OR, odds ratio.

Pooled OR showed a 65% reduced risk of a new rupture episode in the ACLR plus LEAP group compared with the ACLR group: OR 0.35 (95% CI, 0.20-0.59) (*P* < .001). The forest plot in Figure 4 shows the odds ratio for a new episode or rerupture for the 2 groups.

**Figure 5.**
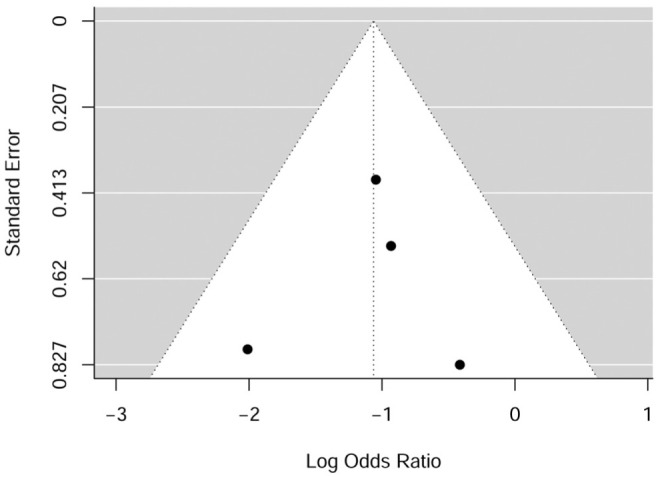
Funnel plot for odds ratio showing the symmetry of the included studies regarding rank correlation and linear regression on rerupture rate.

Neither the rank correlation test nor the linear regression test showed any funnel plot asymmetry (*P* = .750 and .841, respectively). A funnel plot of the estimates is shown in Figure 5.

## Discussion

The most important finding of the current study reveals that in elite athletes, adding an anterolateral procedure during ACLR reduces the rerupture rate and reduces the risk of rerupture by >60%.

Currently, the literature contains considerable debate regarding the treatment of the ACL in the elite athlete—both for graft choice and for whether to add an anterolateral procedure. The current meta-analysis confirms that adding an anterolateral procedure can be a reliable solution to reduce the risk of rerupture.

Elite athletes are more likely to sustain an ACL injury during competition than during practice. The rate of ACL injuries in European professional football matches was found to be 20 times higher than the rate of injuries sustained during practice (0.340 vs 0.017 per 1000 hours). The National Football League witnessed more than twice as many ACL tears during games than during practice over a 10-year span (142 vs 67, respectively). In the National Collegiate Athletic Association, over the course of 5 years, the rate of ACL injury during games for American football was 8.06 per 10,000 athlete exposures, whereas the practice injury rate was 0.8 per 10,000 athlete exposures.^6,14^

In 2018, Lai et al^21^ performed a meta-analysis of estimated graft reruptures in elite athletes and found a rerupture rate of 5.2% in 1272 athletes, which was lower than our finding. Similarly, Walden et al^40^ found that in a 15-year prospective study of elite athletes, the rerupture rate was approximately 4% in 149 players.

Contrasting results were reported in 2021 by Della Villa et al,^11^ who investigated the rate of second ACL injury for 118 players in the Union of European Football Associations (UEFA) Elite Club Injury Study. Almost 1 in 5 top-level professional male football players sustained a second ACL injury after ACLR and returned to football, with a considerably increased risk for players with a noncontact or isolated index injury.

An interesting analysis of professional players was performed by Bloch et al^2^ in 2022. The study analyzed data on ACL injuries among German professional male team sports over 5 consecutive seasons with the aim of improving medical outcomes in the future. Sport-specific differences in injury occurrence, concomitant injuries, timing of ACLR, graft type selection, and short-term complications were examined. The study showed an overall high rate of revision arthroscopy after ACLR (15.6%), which should encourage surgeons and therapists to evaluate their treatment and rehabilitation strategies in this specific subpopulation.

Similar results have been found for amateur athletes. Recently, Mercurio et al^25^ compared the rerupture rate between combined ACLR and ALLR versus isolated ACLR. The rate of graft failure was higher for those with combined ACLR and ALLR (4.6%) compared with those who underwent isolated ACLR (10.4%) (OR, 0.37; 95% CI, 0.18-0.77; *P* = .008). These results completely overlap with those of the current meta-analysis (5.0% for ACLR plus LEAP vs 14.0% for isolated ACLR), confirming that the rate of rupture is reduced in both amateur and elite athletes when LEAP is added to the ACLR.

Since the “rediscovery” of the anterolateral ligament in 2013, much attention has been paid to how the anterolateral structures influence rotatory knee stability. Lateral extra-articular tenodesis is one of various anterolateral extra-articular techniques that have historically been developed to alleviate anterolateral rotational instability.^9^

Previous research, however, raises questions about lateral extra-articular tenodesis’ (LET) nonanatomic nature and possible overconstraint of the joint due to changed kinematics. Furthermore, graft overtensioning may result in excessive restraint, which could ultimately cause further degenerative alterations in the lateral tibiofemoral compartment. ALLR, a relatively new surgery, has been made possible by the recent resurgence of interest in the anterolateral structures of the knee, which has resulted in major advancements in our understanding of anatomy and biomechanics.^12^

Multiple cadaveric studies have analyzed the biomechanical effects of LET procedures, demonstrating that ACLR alone in the presence of an anterolateral injury resulted in residual internal tibial rotation laxity and anterior tibial translation, whereas an additional LEAP was able to restore knee kinematics.^27,28,39,40^

These procedures decrease strain on the ACL graft in addition to increasing rotatory knee stability. In a recent study, the augmentation of ACLR by LET decreased the ACL graft force by 80%. A similar decrease in load on the graft was observed at 30° of knee flexion in a simulated setup.^24^

Recently, van der Wal et al^40^ found that the iliotibial band acts as the main secondary stabilizer to the ACL in resisting internal rotation during the pivot shift and that an anterolateral corner (ALC) reconstruction with either a modified Lemaire tenodesis or an ALLR can improve residual knee rotatory laxity in ACL-reconstructed knees.

The results of cadaveric studies have been consistently confirmed in clinical studies published over the last few years, as shown in our meta-analysis.

In 2024, Bosco et al^5^ analyzed patient-reported outcome measures and ACL reinjury rate after ACLR plus an ALC procedure compared with an isolated ACLR. The systematic review and meta-analysis reported on the importance of combined ACLR with LEAP, including LET and ALLR, in patients with high-grade rotational laxity. Both LEAP techniques—LET and ALLR—were associated with improved pivot-shift tests, better patient-reported outcome measures, and reduced graft failure rates, with no evidence of superiority of one over the other.

Similarly, Park et al^31^ confirmed that ACLR plus ALLR had significantly better outcomes in terms of knee rotational stability and graft failure rate than isolated ACLR.

Earlier, Hurley et al^19^ suggested that concomitant ALLR improves clinical outcomes, with improved knee stability and lower rerupture rates.

Anterolateral procedures improve not only clinical outcomes but also knee stability, which is a key point in elite athletes. In fact, as reported by Kunze et al^20^ in their meta-analysis, contemporary evidence suggests that ALLR improves pivot shift and confers comparable clinical and functional outcomes with isolated ACLR.

At the clinical level, a question that often arises is whether adding extra surgical time may entail risks associated with the procedure. In this regard, D’Ambrosi et al^10^ recently demonstrated how adding LEAP, regardless of the surgical technique, can reduce the failure rate without increasing the number of complications at the midterm follow-up.

### Strengths and Limitations

Several strengths and limitations should be considered in this systematic review and meta-analysis. This study’s power is that it is the first to analyze exclusively comparative studies in elite athletes comparing isolated ACLR versus ACLR combined with LEAP.

Previous systematic reviews and meta-analyses in the literature included older studies with lower levels of evidence and higher risk of bias. The limitations of the current study are related to the limited number of studies with different study designs that we evaluated. The heterogeneity of studies, including ACLR graft selection, LEAP graft selection, attachment points, and surgical techniques, limit direct comparisons when evaluating clinical outcomes. Furthermore, concomitant surgery was not considered an exclusion criterion. Therefore, it is important that future studies specify the length of follow-up, the graft used, and concomitant surgery performed.

## Conclusion

In elite athletes, adding an anterolateral procedure during ACLR significantly reduced the rerupture rate and reduced the risk of rerupture by >60%. Despite the few investigations considered, our study seems to indicate that surgeons should carefully consider LEAP when treating an elite athlete to significantly reduce the risk of rerupture.
